# Structural comparison strengthens the higher-order classification of proteases related to chymotrypsin

**DOI:** 10.1371/journal.pone.0216659

**Published:** 2019-05-17

**Authors:** Heli A. M. Mönttinen, Janne J. Ravantti, Minna M. Poranen

**Affiliations:** 1 Molecular and Integrative Biosciences Research Programme, Faculty of Biological and Environmental Sciences, University of Helsinki, Helsinki, Finland; 2 Applied Tumor Genomics Research Program, Faculty of Medicine, University of Helsinki, Helsinki, Finland; UMR-S1134, INSERM, Université Paris Diderot, INTS, FRANCE

## Abstract

Specific cleavage of proteins by proteases is essential for several cellular, physiological, and viral processes. Chymotrypsin-related proteases that form the PA clan in the MEROPS classification of proteases is one of the largest and most diverse group of proteases. The PA clan comprises serine proteases from bacteria, eukaryotes, archaea, and viruses and chymotrypsin-related cysteine proteases from positive-strand RNA viruses. Despite low amino acid sequence identity, all PA clan proteases share a conserved double β-barrel structure. Using an automated structure-based hierarchical clustering method, we identified a common structural core of 72 amino acid residues for 143 PA clan proteases that represent 12 protein families and 11 subfamilies. The identified core is located around the catalytic site between the two β-barrels and resembles the structures of the smallest PA clan proteases. We constructed a structure-based distance tree derived from the properties of the identified common core. Our structure-based analyses support the current classification of these proteases at the subfamily level and largely at the family level. Structural alignment and structure-based distance trees could thus be used for directing objective classification of PA clan proteases and to strengthen their higher order classification. Our results also indicate that the PA clan proteases of positive-strand RNA viruses are related to cellular heat-shock proteases, which suggests that the exchange of protease genes between viruses and cells might have occurred more than once.

## Introduction

Proteases are a diverse group of enzymes that are required for the cleavage of target proteins in multiple biological processes, such as blood coagulation, complement activation, food digestion, and viral replication [1–3]. A lack of balance in the expression of certain proteases is also associated with cancer development [2, 4], which emphasizes the importance of controlled protease activity for normal cellular function.

Proteases vary in their structural folds and in the composition of the catalytic amino acids. MEROPS is a database and hierarchical classification scheme for proteases [5, 6]. Families in MEROPS are defined as groups of homologous proteins that share significant similarity in amino acid sequence with the peptidase unit of the type example of the family or another protein previously assigned to the family. Families are assigned into a clan if representative family members have clearly similar protein folds. Members of a clan are assumed to share a common origin. If there are clearly distinct groups of proteases within a family and there is evidence of very ancient divergence, the members of a family are divided into subfamilies. One of the most studied protease groups is the chymotrypsin-related proteases that constitute the PA clan in the MEROPS database. The PA clan currently contains nine families of cysteine proteases (representing proteases of positive-strand RNA viruses) and 14 families of serine proteases (representing proteolytic enzymes from eukaryotes, bacteria, some DNA viruses and eukaryotic positive-strand RNA viruses). The cysteine protease family C3 is further divided into eight subfamilies (C3A–C3H); the serine protease families S1 and S39 are divided into six (S1A–S1F) and two (S39A and S39B) subfamilies, respectively.

**Fig 1:**
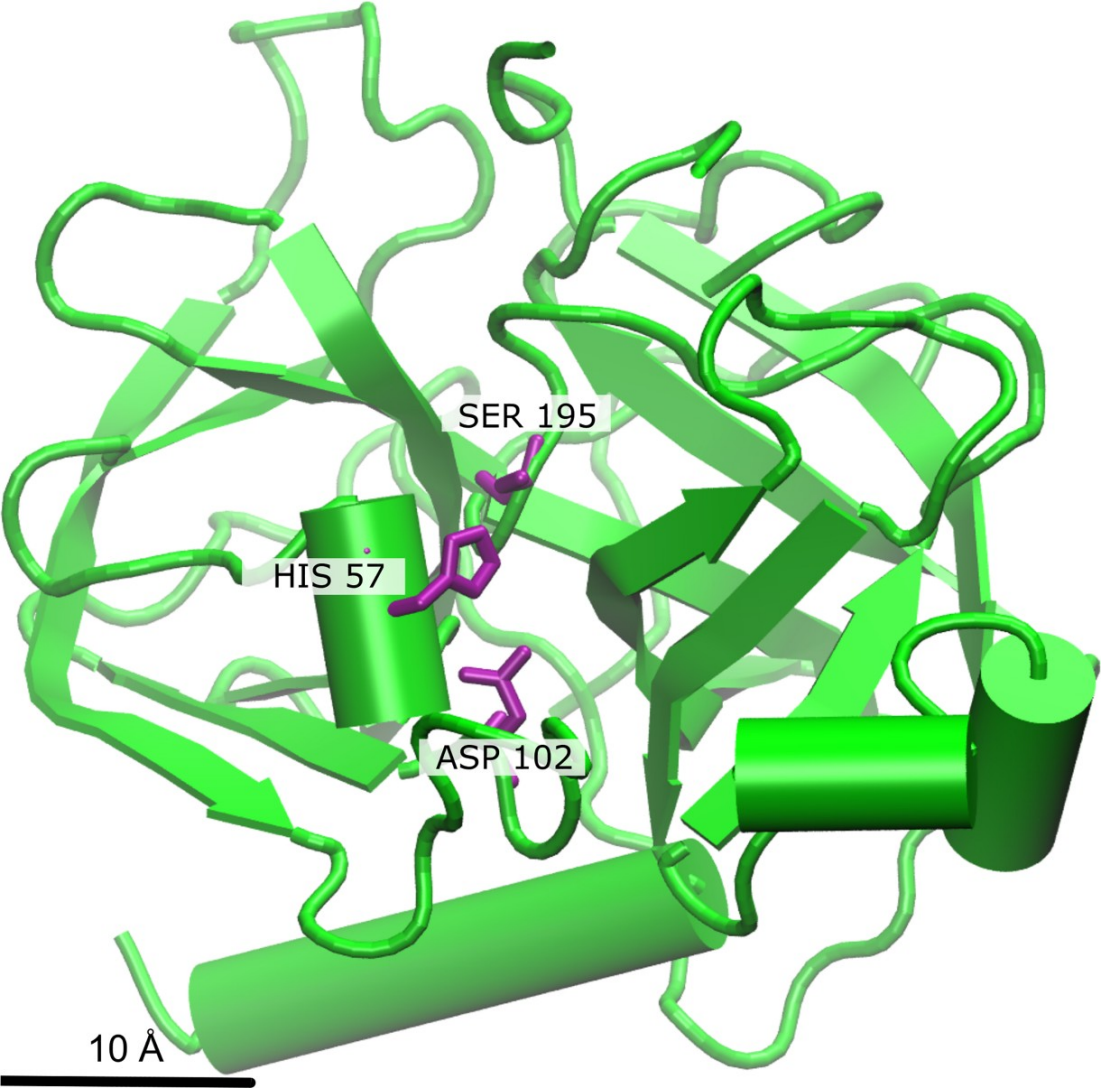
Structural fold of chymotrypsinogen A of *Bos taurus* (PDBid: 2CGA, member of S1A subfamily). The catalytic triad located in the interface of the two β-barrels is shown in purple.

The members of the PA clan proteases share a common structure in which two β-barrel-like domains constitute the catalytic site. The size and completeness of the β-barrels vary. For example, the 2A proteases of enteroviruses (PA clan family C3, subfamily C3B) have only four antiparallel β-strands in place of the N-terminal barrel [7]. The catalytic site is located between the β-barrels and the catalytic triad usually contains His, Asp/Glu, and Ser residues [5, 6] (Fig 1). In cysteine proteases of the PA clan, the triad is composed of His, Asp/Glu, and Cys or of a dyad of His and Cys residues, as in the hepatitis A virus 3C protease and in the coronavirus 3C-like proteases [5, 6].

Experimental structural data is currently available for over 100 PA clan proteases representing 12 protease families. Most of the protein structures are from the S1A subfamily [5, 6], which is also the largest subfamily and includes members from bacteria, eukaryotes, and viruses. The genes encoding the members of the S1A subfamily are extensively duplicated in eukaryotic genomes and have evolved into multiple protease types with diverse functions [8]. Another important group is the viral proteases, which are currently distributed into 20 families within the PA clan. Viral proteases are essential for the cleavage of RNA virus polyproteins (Table 1) [9], but may also enhance the production of viral proteins and inhibit innate host defense mechanisms via cleavage of host translation factors, such as PABP, eIF4G, or eIF5B, as demonstrated for enteroviral 3C and 2A proteases [10–12]. These proteins are expressed during the viral life cycle but are not typically incorporated into the virion (*i*.*e*. they are non-structural proteins). Furthermore, the S1C subfamily (also known as the HtrA family) includes heat-shock proteases activated in response to various stress reactions and is a prominent group among the PA clan proteases. These proteases are present in all the three domains of life and function in multiple roles, such as chaperones and in processes such as protein quality control and stress signaling [13]. Dysfunction of these proteases is associated with diseases such as cancer and Alzheimer’s disease [14].

**Table 1:** Protease families and subfamilies used in this study.

Protease family^a^	Protease subfamily^a^	Number of structures in the study	Type peptidase	Catalytic amino acids	Activity/Function	Organisms
*C3*	C3A	5	C3 protease	His/Asp or Glu/ Cys	Processing of the viral polyprotein, inhibition of host cell protein synthesis.	(+) ssRNA viruses (*Picornaviridae*)
	C3B	2	2A enterovirus peptidase	His/Asp or Glu/ Cys	Processing of the viral polyprotein, inhibition of host cell protein synthesis.	(+) ssRNA viruses (*Picornaviridae*, enterovirus)
	C3C	1	foot-and-mouth disease virus C3 protease	His/Asp or Glu /Cys,	Processing of the viral polyprotein	(+) ssRNA viruses (*Picornaviridae*)
	C3E	1	Hepatitis A C3 protease	His/Asp/Cys (aspartate may not be functional)	Processing of the viral polyprotein	(+) ssRNA viruses (*Picornaviridae*)
*C4*		2	Nuclear-inclusion-a peptidase of plum pox virus	Catalytic triad, His/Asp/Cys	The NIa proteases are required for the processing of the potyviral polyproteins	(+) ssRNA viruses (*Picornaviridae*, potyvirus)
*C30*		7	Porcine transmissible gastroenteritis virus-type main peptidase	Catalytic dyad, His/Cys	Processing of the viral polyprotein	(+) ssRNA viruses (*Coronaviridae*)
*C37*		2	Calicivirin	Catalytic dyad, His/Cys	Processing of the viral polyprotein	(+) ssRNA viruses (*Caliciviridae*)
*S1*	S1A	79	Chymotrypsin A	Catalytic triad, His/Asp/Ser	Many functions, *e*.*g*. intestinal digestion, complement system, blood coagulation and as peptidase in snake venom	Eukaryotes, Bacteria
	S1B	8	Glutamyl peptidase I	Catalytic triad, His/Asp/Ser	*e*.*g*. exotoxins of S*taphylococcus aureus*, highly specific to desmosome	Bacteria
	S1C	8	DegP peptidase	Catalytic triad, His/Asp/Ser	Heat-shock proteases, activated in response to various stress reactions	Bacteria, chloroplasts, and mitochondria
	S1D	4	Lysyl endopeptidase	Catalytic triad, His/Asp/Ser		Bacteria
	S1E	7	Streptogrisin A	Catalytic triad, His/Asp/Ser		Bacteria
	S1F	1	Astrovirus serine peptidase	Catalytic triad, His/Asp/Ser	Processing of the viral polyprotein	(+) ssRNA viruses (*Astroviridae*)
*S3*		3	Togavirin	Catalytic triad, His/Asp/Ser	Capsid protein, cleaves itself from the polyprotein	(+) ssRNA virus (*Togaviridae*)
*S6*		5	IgA1-specific serine peptidase	Catalytic triad, His/Asp/Ser	Interference of mucosal immunity	Bacteria
*S7*		2	Flavivirin	Catalytic triad, His/Asp/Ser	Processing of the viral polyprotein	(+) ssRNA virus (*Flaviviridae*)
*S29*		1	Hepacivirin	Catalytic triad, His/Asp/Ser	Processing of the viral polyprotein	(+) ssRNA virus (*Flaviviridae*, hepacivirus)
*S32*		2	Equine arteritis virus serine peptidase	His/Asp/Ser	Processing of the viral polyprotein	(+) ssRNA virus (*Arteriviridae*)
*S39*	S39A	1	Sobemovirus peptidase	His/Asp/Ser	Processing of the viral polyprotein	(+) ssRNA virus (sobemovirus)
*S46*		2	dipeptidyl-peptidase 7	His/Asp/Ser	Cleavage of peptide for metabolism	Bacteria

^a^According to the MEROPS database. C in the family name indicates cysteine and S serine proteases.

Although members of the PA clan share structural similarity, the amino acid sequence identity between the PA clan families is low. This has significantly hampered the classification of proteases and in some cases the classification was confirmed only after experimentally solving the protein structures (*e*.*g*. the relationship between the serine and cysteine protease members of the PA clan) [15, 16]. The lack of or low level of sequence identity also makes phylogenetic analysis demanding for the PA clan proteases when based solely on the amino acid sequence [15, 16]. Thus, the PA clan proteases are an ideal group for investigation using structure-based methods.

In this study, we applied automatic structure alignment and the structure-based classification method Homologous Structure Finder (HSF) [17] to re-evaluate the relationships within and between the families of the PA clan. HSF identifies the equivalent residues for a pair of protein structures by comparing a set of amino acid properties (*e*.*g*. physiochemical properties of amino acids, local geometry, backbone direction, local alignment, and Cα distances) [17]. The two protein structures that are the most similar based on the properties are merged into a common structural core which then represents the pair in the later iterations. The iteration is continued until all the protein structures are part of a clustering and a single structural core is identified for all the proteins in the data set. The equivalent residues in the structural core can be considered homologous, similar to high-scoring columns of multiple sequence alignment. A pairwise comparison of the properties of the residues in the homologous positions of the common structural core between the original structures results in a pairwise distance matrix, which can be used for constructing a structure-based distance tree [17]. The distances in such structure-based distance trees do not necessarily reflect exact evolutionary distances, as changes in protein structure may not be continuous. However, the clustering of proteins in the structure-based distance tree constructed using HSF has been shown to follow the sequence-based classification of proteins into protein families, even when the common core contains less than 40 residues [18, 19]. Thus, structure-based analysis is appropriate for a rough estimation of evolutionary events and relationships between protein families when the proteins share little or no detectable sequence similarity.

The main limitation of HSF and other structure-based approaches is the biased sampling of high-resolution structures in the databases. However, recent developments in the field of structural biology have significantly increased the number of new protein structures and facilitated studies on different proteins and protein complexes. Therefore, it is important to develop structure-based protein comparison methods to complement sequence-based approaches.

Previous studies have identified highly superimposable structural regions at close proximity of the catalytic site among the members of the S1 family of the PA clan [20]. Here, we describe a common structural core of 72 residues for proteases, representing 12 different families of the clan. We then derived a structure-based distance tree based on the identified core. To our knowledge, this is the first attempt to comprehensively study the relationships of the PA clan protease families. The structure-based distance tree precisely follows the established protease subfamilies, although the core does not contain any unique subfamily-specific features. Notably, this structure-based distance tree more precisely follows the MEROPS classification than the sequence-based phylogeny deduced for the same set of proteases. Structure-based distance analyses could thus be used to complement sequence-based methods in the systematic classification of proteins, particularly when sequence similarity is minimal and the alignment region is short. Moreover, our results support the earlier conclusions that the PA clan proteases of RNA viruses are related to the cellular heat-shock proteases of subfamily S1C (*i*.*e*. HtRA proteases) [15, 21]. In addition, our results indicate that the exchange of protease genes between viruses and cellular organisms may have occurred more than once.

## Materials and methods

### Selection of protein structures

Protein structures for the analysis were selected from the Protein Data Bank (PDB) (www.pdb.org; structures published before 11 February 2016; see S1 Table) by selecting one protein structure from each protease family and subfamily of the PA clan defined in the MEROPS database (https://www.ebi.ac.uk/merops) [5, 6]. These structures were subsequently used for DALI searches [22] (ekhidna.biocenter.helsinki.fi/dali_server) to enlarge the data set. To assure that the chosen protein structures were large enough to contain both β-barrels, only protein structures containing ≥138 amino acids were used for further analysis. The resulting dataset was filtered such that amino acid sequences of the protein structures represented pairwise sequence identity of 70% at maximum. Filtering was performed by using CD-hit [23, 24]. The protein structures of the resulting dataset were manually verified and some structures were replaced if a higher quality protein structure was available. The criteria for replacing a protein structure were: 1) a more complete structure in the catalytic region, 2) fewer amino acid substitutions, and 3) higher resolution. In addition, the structures of the S6 protease family (see S1 Table) were cut such that only the serine protease domain remained; this prevented the other domains from interfering in the structure alignment.

### Structural alignment and identification of common cores

The equivalent residues between the protein structures (*i*.*e*. the common core) were identified by using HSF [17–19]. Parameters optimized for right-hand-shaped polymerases described in Mönttinen et al. [18] were initially used. This optimization was performed using a self-written Python script. Further optimization was specifically performed for the following three parameters: amino acid type, local geometry, and cut-off distance between the equivalent Cα-residues. The values for these parameters were manually selected based on those that resulted in the proper alignment of the corresponding β-barrels between structures and yielded the lowest average root-mean-square deviation (rmsd) and largest number of equivalent residues (see S2 Table). The Visual Molecular Dynamics 1.9.2. program was used for the visualization of the protein structures and structural cores [25].

### Validation of the results using DALI searches

DALI [22] is a well-established method and tool for pairwise comparisons of protein structures. DALI searches for viral proteases were performed to identify the structurally most similar cellular structures (structures published before 29 April 2017). In addition, DALI searches on S1D subfamily proteases (structures published before 15^th^ of April 2018) were performed to validate the division of the S1D subfamily into two groups.

### Sequence alignments and sequence-based phylogenetic analysis

The amino acid sequences of the selected protein structures were downloaded from PDB (www.pdb.org) and were aligned using Mafft v7.146b with E-INS-I parameter [26, 27]. The alignment was trimmed using trimAl [28] with the “gappyout” parameter (see the alignment in S1 Appendix). The phylogeny was made using iqtree [29] with automated ModelFinder [30] and ultrafast bootstrap [31] options. The substitution model used was WAG+R6 [32, 33].

The pairwise sequence alignments were performed using Smith-Waterman algorithm [34].

### Structure-based distance trees

Structure-based distance trees were constructed by comparing the identified sets of equivalent residues. The branch lengths of the trees were calculated as described [17]. The normalized distance-matrix was converted to a tree by using the Fitch-Margoliash algorithm that is applicable to structure-based trees [35]. The structure-based distance trees were visualized using Dendroscope 3.4.4 [36].

To evaluate the robustness of the structure-based distance tree, a simplified jackknife test was performed as described previously [19]. A single structure from each protease subfamily/family was discarded one by one and a structural core was identified for the remaining 142 protein structures. A new structure-based distance tree was calculated based on this structural core. A simplified jackknife test was used due to the relatively high computational requirements of the structural alignment method.

### Comparison of interaction energies

To evaluate the structural stability of the identified core, and the stability of regions outside the core, we calculated pairwise interaction energies for all the amino acid residue pairs in selected members of each family/subfamily using the Interaction Energy Matrix Web Application (http://took87.ics.muni.cz:8080/energy2/) [37]. The applied parameters were CHARMM36 [38] for force field, solvent for environment, and ADD for hydrogens parameter. The amino acids that are stabilizing for a protein structure receive negative values (kJ/mol) [37, 39]. The means of interaction energies were calculated separately for two sets: 1) amino acid residue pairs belonging to the core, and 2) amino acid residue pairs not belonging to the core. The significance of difference in interaction energies between the core and non-core amino acids was deduced by calculating p-values using Mann-Whitney U test.

## Results and discussion

### Protein structures of PA clan proteases

The protein structure data set was collected from the PDB by selecting a single representative structure from each protease family/subfamily of the PA clan (MEROPS database [5, 6]) for which structural information was available. These structures were then used for a DALI search [22]. The resulting data set was filtered such that the selected structures shared at the most 70% amino acid sequence identity. This filtering was performed as highly similar structures would not provide additional information about the relationships between families and subfamilies but would notably increase the computation time. After filtering, the data set was further manually curated. Structures were removed if they lacked a complete catalytic site with two β-barrel-like domains; there were more than 10 missing residues per structure; or the resolution of the protein structure was >4.0Å. Some initially selected protein structures were also replaced if a higher-quality protein structure (according to criteria above) was available in the original cluster of 70% sequence identity (see Materials and Methods). The minimum and median amino acid sequence identity values between pairs of selected proteases were 0.1% and 15.0%, respectively (using Smith-Waterman algorithm [34]).

The resulting data set contained 143 protease structures, representing 12 families and 11 subfamilies of the PA clan (Table 1, see S1 Table). The structures were found from eukaryotes, eukaryotic organelles, bacteria, and positive-strand RNA viruses. The data set had four cysteine and eight serine protease families (Table 1). The cysteine proteases from families C4, C30, C37, and C3 (including subfamilies C3A, C3B, C3C, and C3E) are all from positive-strand RNA viruses. The serine protease families S6 and S46 and subfamilies S1B, S1C, S1D, and S1E of the S1 family comprise proteases from bacteria and eukaryotic cell organelles. The available protease structures of subfamily S1A of S1 serine proteases were all from eukaryotes. The selected positive-strand RNA virus serine proteases were from families S1 (subfamily S1F), S3, S7, S29, S32, and S39 (subfamily S39A).

### The common structural core of the PA protease clan

The 143 selected PA clan protease structures were structurally aligned using HSF. The alignment is based on several parameters, such as amino acid sequence, secondary structure, geometry, and physiochemical properties of the amino acids (see [17]). This results in the identification of equivalent residues between protein structures and the identification of a common structural core for a set of protein structures. The final optimized parameters (S2 Table) used here for structural alignment were adjusted from those previously used for right-hand-shaped polymerases and structurally related enzymes [18, 19] (see Materials and Methods).

**Fig 2:**
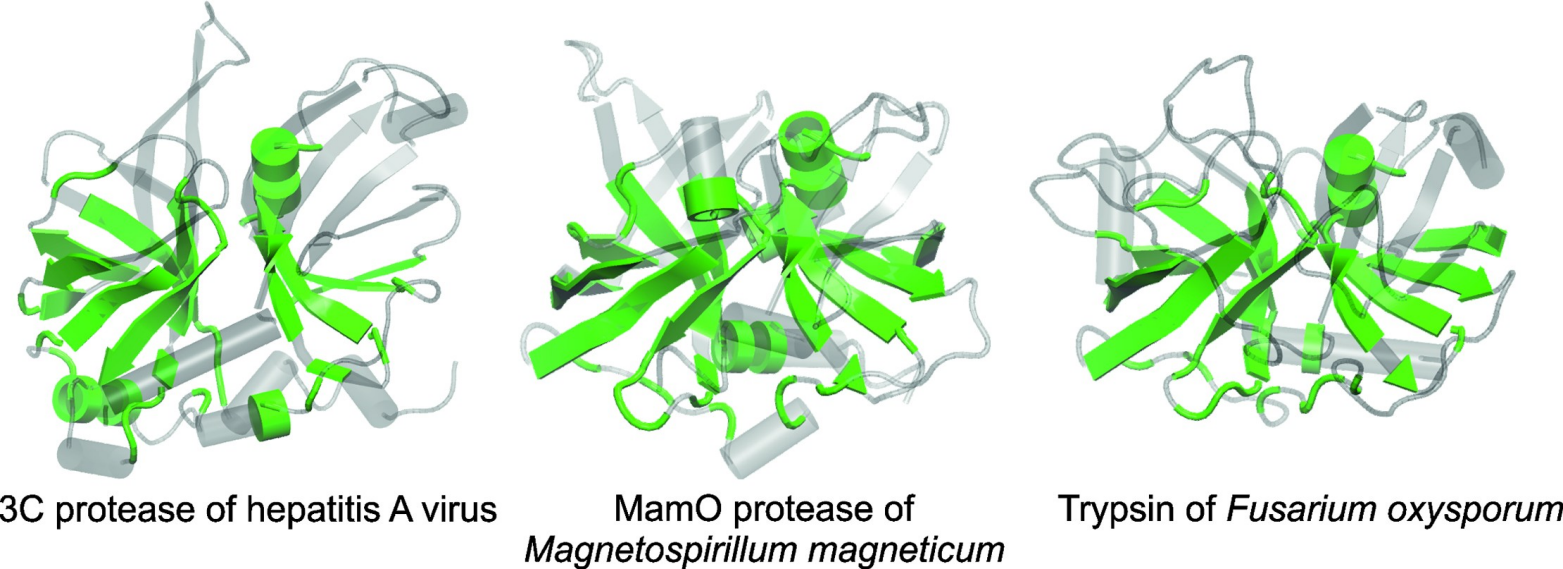
Common structural fold for PA clan proteases. The common structural core for the PA clan proteases was identified using the HSF program. The 72 equivalent residues deduced from the structural clustering are mapped in green on the structures of the 3C protease of the hepatitis A virus (left; family C3, subfamily C3E, PDBid: 1HAV), MamO protease of *Magnetospirillum magneticum* (middle; family S1, subfamily S1C, PDBid: 5HMA), and trypsin of *Fusarium oxysporum* (right; family S1A, PDBid: 1GDQ). The other parts of the protein structures are shown in grey.

**Fig 3:**
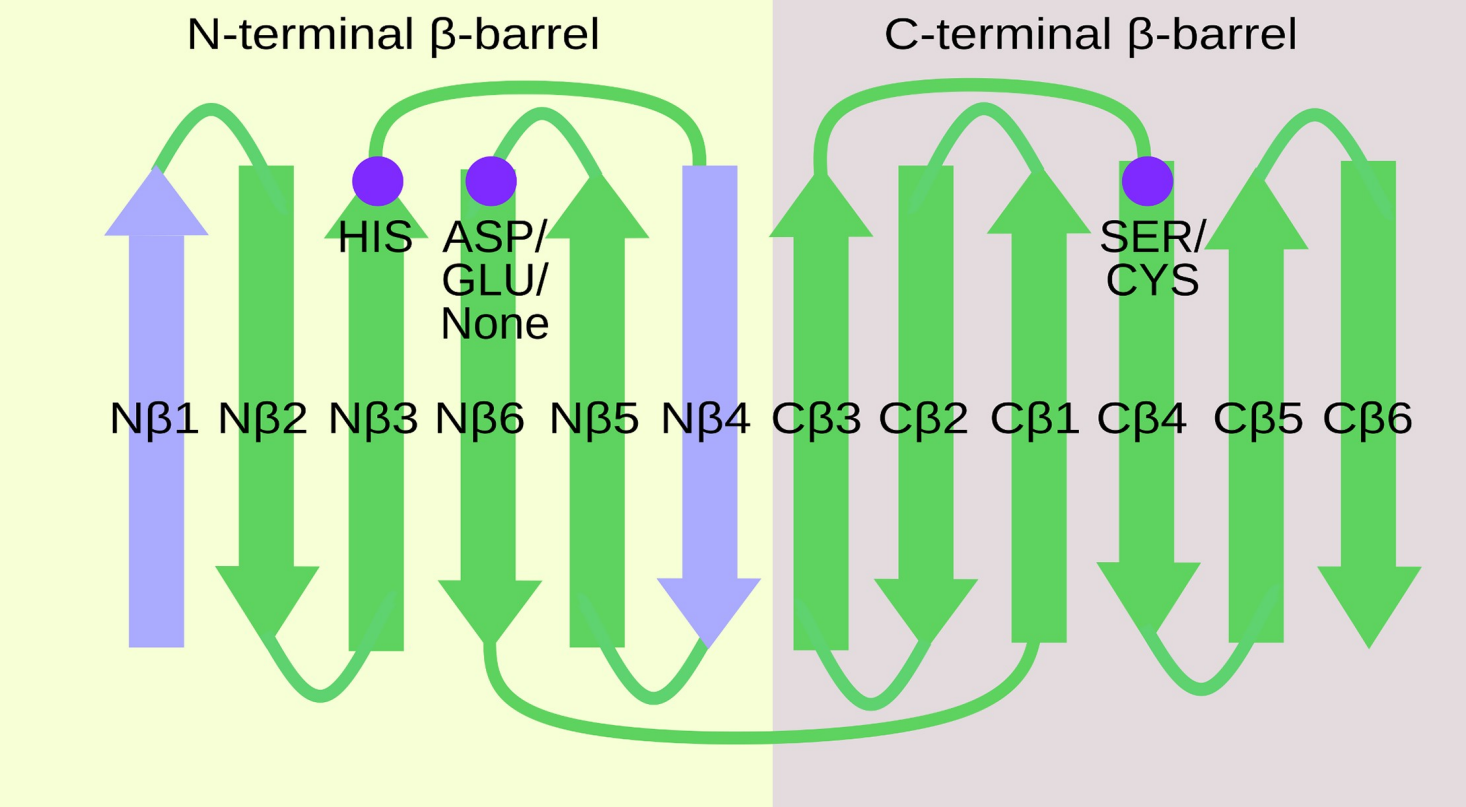
Cartoon of the secondary structures in the canonical two β-barrel structure of PA clan proteases and the identified structurally conserved core. The secondary structures observed in the identified core are shown in green. Elements observed only in the canonical β-barrel structure are shown in light purple. The catalytic amino acids are indicated with dark purple spheres (positions according to trypsin). The secondary structures are numbered. N-terminal secondary structures start with N and C-terminal with C.

Through the iterations, HSF identified a common structural core of 72 residues with an average rmsd of 2.2Å for all the PA clan proteases in the data set. The equivalent residues were located mainly at the interface of the two β-barrel domains forming the catalytic site. This is depicted for three distinct PA clan members in Fig 2 (for the catalytic residues see also Figs 1 and 3). The identified core lacks β1- and β4-strands of the canonical N-terminal β-barrel (Fig 3). The size and the general similarity (low rmsd) of the protease core indicates that the structural fold of proteases, especially at the catalytic site, is under strong natural selection [40, 41].

The identified core resembles some of the smallest members of the PA clan, such as the 2A protease of rhinovirus (subfamily C3B), in which the N-terminal β-barrel comprises only four β-strands [6]. The catalytic amino acids are located in the third and sixth β-strands of the N-terminal β-barrel (His and Asp/Glu, respectively) and in the fourth β-strand of the C-terminal β-barrel (Ser or Cys) (see Fig 3). In addition to the catalytic amino acids, the surrounding residues participate in stabilization of the triad via H bonds [42]. Calculation of interaction energies for the identified core region and regions outside of the core from a representative structure of each protease family/subfamily included in this study revealed that the core region in all of the selected proteases has a lower average interaction energy between its residues than the rest of the structure. The calculated average interaction energies within the core were approximately 2.7 times lower than the calculated average interaction energies of the other regions of the protein. This indicates that the core residues are important for stabilizing and maintaining the overall structure of the protein (S3 Table). The extensions and loops between the β-strands of the N- and C-terminal β-barrels are not shared by all the members of the PA clan and are thus not present in the identified core structure. This extension and these loops are typically required for more specific functions of the protease, such as recognition and binding of ligands (*e*.*g*. exosite I of thrombin binds a cofactor [43]). Thus, the identified structurally conserved core likely represents the minimum structure to perform the catalytic reaction, while the regions outside the core are adaptations to the specific environment and function of the protease.

### Relationships within the PA protease clan

#### Construction and validation of the structure-based distance tree

**Fig 4:**
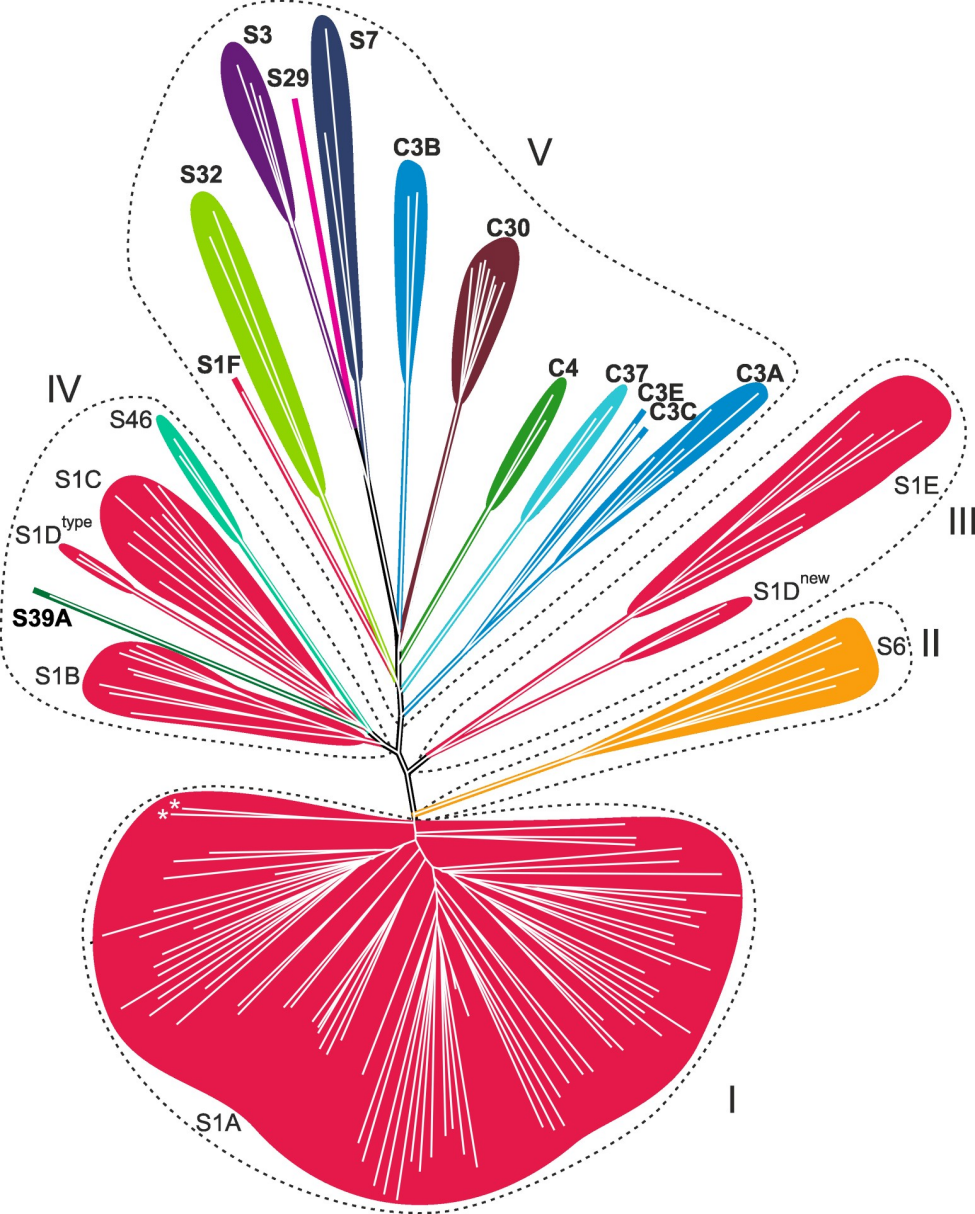
A structure-based distance tree for members of PA clan proteases. The structure-based distance tree was deduced based on the 72 equivalent amino acid residues located close to the catalytic site. The colors indicate the different families of the PA clan according to the MEROPS database. The five clusters (I−V) are indicated. The split subfamily S1D groups are labeled with “S1D^type^” and “S1D^new^”. The branches corresponding to the protease paralogs SMIPP-S-D1 and SMIPP-S-I1 (PDBids: 3H7T and 3H7O, respectively) are marked with asterisks. The names of the families/subfamilies that comprise viral proteases are in bold.

A structure-based distance tree was calculated based on the 72 residues forming the common structural core of the PA clan proteases. The resulting tree revealed that the families/subfamilies of PA clan are roughly clustered into five groups (from I to V; Fig 4) as discussed below. The robustness of this clustering was tested with a simplified jackknife test suitable for structure-based distance trees [19]. In this test, a member from each subfamily/family is discarded one at a time and a new structure-based distance tree is repeatedly calculated using the remaining dataset (here 142 structures; S1 Fig). This analysis confirmed that the outline of the structure-based distance tree presented in Fig 4 is robust at the group and MEROPS subfamily levels (compare Fig 4 to S1 Fig). The only exceptions are the viral proteases of subfamily S32, which clustered in 15% of replicates with group IV and in 85% of replicates with group V. Interestingly, the members of the S32 subfamily also received the highest scores in the initial DALI searches with the members of either groups IV or V (S4 Table).

#### Clustering of PA proteases in the structure-based distance tree follows the subfamilies of MEROPS classification

The clustering of PA proteases in the structure-based distance tree was based on the identified common structural core, which does not cover regions previously considered characteristic for each subfamily [44]. Nevertheless, the obtained clustering follows the MEROPS classification at the subfamily level (Fig 4). The only exception is the subfamily S1D, which is split into two groups. This division was also maintained in the simplified jackknife test (see S1 Fig), suggesting that division of subfamily S1D into two separate subfamilies could be considered. Here, we have used subfamily names “S1D^type^” and “S1D^new^” to indicate these two groups (Fig 4). The first one includes *Achromobacter* protease I (PDBid: 1ARB) and the type example of the current S1D subfamily lysyl endopeptidase of *Lysobacter enzymogenes* (PDBid: 4NSY). The second (S1D^new^) contains the thermostable serine protease AL20 of *Nesterenkonia abyssinica* (PDBid: 3CP7) and the Anisep protease from *Arthrobacter nicotovorans* (PDBid: 3WY8). The S1D^type^ group is clustered with the S1B and S1C subfamilies and this clustering is also maintained in all the replicates of the simplified jackknife test (S1 Fig). The S1D^new^ group clusters with members of the protease subfamily S1E, and this clustering was observed in 75% of the replicate runs (see S1 Fig). DALI results also support the division of S1D into two subgroups; members of both subgroups received the best hits within the new subgroups. However, the Z-score similarities, rmsd values, and sequence identities between members of different subgroups were comparable to those obtained when S1D proteases were compared to the other subfamilies of the S1 family (DALI search on 15 April 2018). The proteases in the identified S1D^type^and S1D^new^ are from distantly related bacterial phyla, namely *Proteobacteria* and *Actinobacteria*, respectively. The distinct evolutionary history of these bacteria could at least partially explain the observed structural diversification of the S1D proteases into two groups.

Notably, the structural clustering followed the established MEROPS subfamilies more precisely than the amino acid sequence-based phylogeny made for comparison using the same set of proteases (compare Fig 4 to S2 Fig). This observation suggests that analysis of proteins that share a low overall sequence similarity and only short aligning regions may benefit from structure-based analysis.

#### Identification of subgroups within the S1A subfamily

The S1A subfamily proteases form a clearly distinct group in the structure-based distance tree (group I; Fig 4). However, two of its members, the protease paralogs SMIPP-S-D1 and SMIPP-S-I1 of *Pichia pastoris* (PDBids: 3H7T and 3H7O, respectively), were clustered apart from the other members of the S1A subfamily (Fig 4). These two paralogs are not functional proteases [45], which explains their loose connectivity to the other members of the S1A subfamily and underlines how functional diversification drives the structural evolution of homologous proteins.

#### Clustering of PA clan subfamilies into families in the structure-based distance tree

Two of the studied protease families (S1 and C3) contained more than one subfamily and thus allowed evaluation of the family-level clustering. Three out of four subfamilies of the C3 family (C3A, C3C, and C3E) formed a stable cluster in the structure-based distance tree, whereas subfamily C3B was clustered separately in 95% of the replicates (see Fig 4 and S1 Fig). Subfamily C3B includes picornaviral 2A proteases, which have a slightly different function compared to the other members of the C3 protease family. This functional conversion has led to a different structural evolutionary trajectory, which has materialized as a partial deletion of the N-terminal β-barrel [5, 6] among the subfamily C3B members.

The six subfamilies of S1 (Table 1) were located close to each other in the structure-based distance tree (Fig 4 and S1 Fig), primarily in groups I, III, and IV. However, in groups IV and V, the S1 family members were clustered together with the members of other PA clan families. In group IV, the members of subfamilies S1B (bacterial proteases), S1C (bacterial and cell organelle proteases), and two members of the S1D^type^ group (bacterial proteases) formed a robust cluster together with the bacterial proteases of the S46 family and the S39A subfamily proteases of positive-strand RNA virus. The higher structural similarity of the S39A protease to cellular than to viral proteases has also been previously reported [46]. In group V, the representative structure of the S1F subfamily (Astrovirus serine peptidase) is grouped together with members of subfamily S32 within a large cluster of other cysteine and serine proteases of positive-strand RNA viruses (Fig 4 and S1 Fig). Furthermore, the S6 family forms an independent group II. This group is located between groups I and III that both contain only members of the S1 family. The members of S6 family are autotransporter proteins in gram-negative bacteria; all the members of S6 family proteases have a long β-stalk structure at the C-terminus, which was not found from any other PA clan proteases [47]. Despite the large additional domain and the low sequence identity, the S6 serine protease domain structurally resembles members of the S1A subfamily the most [47], thus supporting the location of the S6 branch within the S1 subfamilies. Based on previous observations [46, 47] and the data presented here, the families S6 and S46 and the subfamily S39A could be considered as part of the S1 family.

#### The PA clan serine and cysteine proteases of positive-strand RNA viruses (group V) are related to serine proteases of group IV

Within the structure-based distance tree (Fig 4), all the cysteine and serine proteases of positive-strand RNA viruses except that of sobemovirus serine protease (subfamily S39A) were clustered together (group V) apart from all cellular proteases. The high mutation rates of RNA viruses compared to cellular organisms can deteriorate the detectable signal of sequence similarity between homologous cellular and viral proteins, thus making it difficult to trace their relationships. However, in our structure-based analyses, group V of viral PA clan proteases was always located in close proximity to the group IV proteases (Fig 4 and S1 Fig), indicating a common origin for these two groups of proteases. Group IV contains eukaryotic and bacterial HtrA proteases of subfamily S1C and bacterial serine proteases of subfamilies S1B and S46 and the S1D^type^ group (such as dipeptidyl-peptidases, lysyl endopeptidase, glutamyl endopeptidase I, and SplA peptidase). Exchanges of protease genes between eukaryotic viruses and their hosts likely explains the observed structural relatedness of the abundant eukaryotic HtrA proteases (belonging to the S1C subfamily) and the viral proteases of group V. However, intracellular bacteria of eukaryotic cells (such as *Mycobacterium tuberculosis*) also have HtrA protease genes, thus offering an alternate gene transfer route for PA proteases of positive-strand RNA viruses and HtrA proteases [5, 6]. In the initial DALI searches for the viral PA clan proteases, the highest scoring cellular proteases in 9 cases out of 13 were among members of the subfamily S1C of group IV (S4 Table). In addition, representatives of two viral protease subfamilies (C3B and S39A) achieved the best hits for cellular proteases outside the S1C group. Thus, the closest cellular relatives for the known viral PA proteases seem to be among the members of the S1C family. This hypothesis is supported by previous studies (based on amino acid sequence or structure-based comparisons), which indicated that viral 3C proteases might have evolved from the HtrA family [15, 21]. In addition, our results suggest that the lateral gene transfer between the cellular protease genes of group IV and the genomes of positive-strand RNA viruses has occurred more than once. This is demonstrated by the observation that the serine protease of sobemovirus in group IV (S39A subfamily) is located separately from all the other viral proteases clustered in the group V (see Fig 4). In previous studies on viral proteases, the sobemovirus protease has also appeared to be only distantly related to the proteases of picornaviruses and secoviruses [15].

#### Relationships between families of viral proteases

Group V contains eight viral protease families of the PA clan. Of these families, those consisting of flavivirus (families S29 and S7) and togavirus (family S3) proteases were always clustered together in the structure-based distance tree as sister groups (see Fig 4 and S1 Fig), even though these viruses belong to different viral families (*Flaviviridae* and *Togaviridae*).

Togavirin is a protein of alphaviruses (members of the *Togaviridae* family), which consists of an N-terminal RNA binding region and a C-terminal region comprising the PA-clan protease. The proteases of positive-strand RNA viruses are typically so-called non-structural proteins, (*i*.*e*. they are not structural components of the virion). However, togavirin is not only a viral protease but also serves as the major capsid protein of the virus [48]. Previously, it was observed that togavirin is structurally similar to flavivirus protease NS3 (protease family S29). It was proposed that togavirin originates from a non-structural viral protease that replaced the coat protein in alphaviruses [48, 49]. Our results support the close relationship between these proteases. However, from our analyses it is impossible to deduce the direction of gene transfer between the different viruses. Nevertheless, the unique capsid protein function of togavirin among all the known PA clan proteases suggests that togavirin likely originates from the non-structural proteases of flaviviruses.

The positions of the remaining viral protease families within group V were not stable in the constructed structure-based distance trees (S1 Fig), which likely reflects the relatively low number of viral proteases, the generally high variability of viral sequences, and the lack of close relatives of these proteases in this data set. Nevertheless, the clustering of viral proteases largely followed the classification of viruses into viral families. The only exception was the picornavirus C3B proteases, which clustered separately from the other picornavirus proteases.

## Conclusions

We have applied an automated structural alignment and clustering method to the PA clan proteases. We identified a common core of 72 structurally equivalent residues at the active site of these proteases (Fig 2 and Fig 3). By comparing this conserved region, we deduced a structure-based phylogenetic tree for the PA clan proteases (Fig 4), which confirmed the established classification at the subfamily level with only one exception. The previously assigned S1D subfamily was split into two distinct groups, which we referred to as S1D^type^ and S1D^new^ (Fig 4).

We have previously shown that even relatively small conserved protein substructures (“common cores”) can be used to define interfamily and even intersuperfamily relations, extending the evolutionary timeframe of protein phylogenies [19]. In this work, the identified core was substantially larger (>2×) and structurally less variable (average rmsd 2.2Å versus 3.6Å) than in the previous study [19]. The better-defined core increased the accuracy of the method, demonstrated by the robustness of the constructed phylogenic tree (S1 Fig). The obtained higher order grouping of proteases (Fig 4) mainly followed the previously proposed protease subfamilies [5, 6]. The agreement between the MEROPS classification and our clustering analysis suggests that structural clustering could be used as an ancillary tool for objective classification of proteins when structural information is available for a representative set of proteins assigned to a clan.

Our results show that structure-based approaches can complement sequence-based analyses at the subfamily level and facilitate the higher order classification of proteins, extending the evolutionary timeframe of current protein phylogenies. Utilization of structural information is especially useful when the signal from the sequence similarities is weak, such as when relationships within diverse and ancient protein clans (like the PA proteases) are evaluated.

Viral enzymes, such as proteases, are important targets for antiviral therapies and there are several protease inhibitors currently in clinical use. The identification of structural conservation in viral proteases may facilitate development of broad-spectrum antivirals that target different single-stranded RNA viruses.

## Supporting information

S1 Table Protein structures used in this study. (XLSX)

S2 Table HSF parameters and applied values. (DOCX)

S3 Table Interaction energies between core residues and between non-core residues for representative PA clan protease structures. (PDF)

S4 Table Best-hit cellular proteases from DALI search on viruses. (PDF)

S1 Fig Replicates of jackknife tests. Replicates of jackknife tests to determine the effects of dataset on overall topology of the structure-based distance tree. The protein family which member has been removed, the resulting core size and the resolution of the alignment are indicated under each tree. The colors indicate the PA clan families as in Fig 4.
(PDF)

S2 Fig Amino acid sequence-based phylogenetic tree for PA clan proteases. The branches are merged and colored according to the protease family as in Fig 4. The branches are marked with a black dot if the ultrafast bootstrap value is ≥0.95.
(TIFF)

S1 Appendix Amino acid sequence alignment of proteases used in this study. (TXT)
