# Supplementary material for: Structural comparison strengthens the higher-order classification of proteases related to chymotrypsin
Source: PLoS One. 2019 May 17;14(5):e0216659. doi: 10.1371/journal.pone.0216659 (PMC6524800; doi:10.1371/journal.pone.0216659)
Supplement: S2 Table — (DOCX) [file pone.0216659.s002.docx]

**S2 Table. HSF parameters and applied values**

| Property |  |  | Weight  (Scaled to 100) |  |
| --- | --- | --- | --- | --- |
| Geometry |  |  |  |  |
|  | Half sphere exposure (HSE) Cα-Cβ |  | 3.585 |  |
|  | HSE down |  | 3.341 |  |
|  | HSE up |  | 2.940 |  |
|  | Dihedral angular φ |  | 3.169 |  |
|  | Dihedral angular ψ |  | 3.384 |  |
|  | Distance to center of gravity |  | 1.119 |  |
|  | Density |  | 3.441 |  |
|  | Moment of inertia |  | 1.090 |  |
|  | Local geometry^1^ |  | 21.696 |  |
| Secondary structure |  |  |  |  |
|  | Secondary structure type |  | 2.538 |  |
|  | Position in secondary structure |  | 2.882 |  |
| Sequence |  |  |  |  |
|  | Amino acid type |  | 2.246 |  |
|  | Position in sequence |  | 3.441 |  |
| Physicochemical properties of amino acids |  |  |  |  |
|  | Hydroxylic |  | 1.190 |  |
|  | Hydrophobic |  | 2.438 |  |
|  | Aliphatic |  | 1.377 |  |
|  | Aromatic |  | 1.133 |  |
|  | Charged |  | 1.391 |  |
|  | Negative |  | 1.176 |  |
|  | Positive |  | 1.162 |  |
|  | Polar |  | 1.965 |  |
|  | Small |  | 1.864 |  |
|  | Tiny |  | 1.262 |  |
|  | Turn-like |  | 2.452 |  |
| Variable |  |  |  |  |
|  | Cα-Cα distance |  | 5.535 |  |
|  | Backbone direction |  | 1.276 |  |
| Other |  |  |  |  |
|  | Local alignment^2^ |  | 20.965 |  |

^1^Local geometry is defined on a ±4 residue-window around a given residue. The similarity is defined as a sum of differences of distance matrices calculated from Cα-coordinates of each window within each pair of residues from each compared core.

^2^Dynamic programming module is used to further detect local agreements of the similarities (e.g. improving alignment of helices between cores). The module produces a new reweighted residue similarity matrix from the equivalences according to given parameters (match, mismatch and gap).
