# Supplementary material for: Structural comparison strengthens the higher-order classification of proteases related to chymotrypsin
Source: PLoS One. 2019 May 17;14(5):e0216659. doi: 10.1371/journal.pone.0216659 (PMC6524800; doi:10.1371/journal.pone.0216659)
Supplement: S3 Table — (PDF) [file pone.0216659.s003.pdf]

S3 Table. Interaction energies between core residues and between non-core residues for representative PA clan protease structures

Protease family/ subfamily	Protein Data Bank id and chain	Mean of interaction energies between core residues (kJ/mol)	Mean of interaction energies between non-core residues (kJ/mol)	A ratio core/non-core	p-value**
C3A	2XYA A	-0.907894757	-0.459704223	2.0	1.10E-55
C3B	2HRV A	-0.927752153	-0.548631793	1.7	1.15E-17
C3C	2WV4	-0.944014925	-0.372575409	2.5	4.42E-64
C3E	1HAV A	-0.881520548	-0.356842709	2.5	1.92E-66
C4	1LVM A	-1.049918168	-0.331230253	3.2	8.19E-92
C30	1P9U D	-0.923996869	-0.268007395	3.4	5.74E-139
C37	2IPH A	-0.976931898	-0.385478517	2.5	4.68E-59
S1A	2XXL A	-0.956851214	-0.247814548	3.9	3.81E-167
S1B	1QTF A	-0.892691706	-0.299247041	3.0	3.88E-102
S1C	1L1J A	-0.716092085	-0.235799862	3.0	5.73E-58
"S1D^{type}"	1ARB A	-0.902734926	-0.332529392	2.7	1.40E-101
"S1D^{new}"	3CP7 A	-0.952379851	-0.417241641	2.3	1.72E-67
S1E	2SFA A	-0.904409785	-0.430233509	2.1	1.15E-53
S1F	2W5E A	-0.916716586	-0.48549025	1.9	5.80E-28
S3	1EP5 A	-0.872177373	-0.519585675	1.7	1.30E-32
S6	1WXR A*	-0.480252743	-0.256131423	1.9	2.14E-07
S7	2M9P A	-0.758945576	-0.246600856	3.1	5.72E-76
S29	2FM2 A	-0.877923951	-0.354291447	2.5	1.46E-53
S32	1MBM A	-0.813202036	-0.399996569	2.0	1.38E-52
S39	1ZYO A	-0.936883959	-0.4576339	2.0	2.25E-47
S46	3WON A	-0.918613547	-0.148145847	6.2	9.84E-258

* Protease domain of 1WXR A.

** To test if interaction energies between core residues are significantly lower than interaction energies between non-core residues
