# Supplementary material for: Structural comparison strengthens the higher-order classification of proteases related to chymotrypsin
Source: PLoS One. 2019 May 17;14(5):e0216659. doi: 10.1371/journal.pone.0216659 (PMC6524800; doi:10.1371/journal.pone.0216659)
Supplement: S4 Table — (PDF) [file pone.0216659.s004.pdf]

S4 Table. Best hitting cellular proteases from DALI search to viruses

**Virus protease**			**Best hitting cellular protease from DALI search**				
Protease family/ subfamily	PDBid and chain	Virus	PDBid	Name	Organism	Protease family	Z-score
C30	1P9U A	transmissible gastroenteritis virus	1DUE A	exfoliative toxin A	*Staphylococcus aureus*	S1B	13.4
C37	2IPH A	Norwalk virus	5ILB A	protease Do-like 2	*Arabidopsis thaliana*	S1C	13.9
C3A	2XYA A	rhinovirus	3TJN A	serine protease HTRA1	*Homo sapiens*	S1C	16.0
C3B	2HRV A	rhinovirus	4SGB E	proteinase A	*Streptomyces griseus*	S1E	13.0
C3C	2VW4 A	foot-and-mouth disease virus	3QO6 B	protease Do-like 1	*Arabidopsis thaliana*	S1C	17.9
C3E	1HAV A	hepatitis A virus	3QO6 C	protease Do-like 1	*Araidopsis thaliana*	S1C	16.3
C4	1LVM A	tobacco etch virus	4YNN F	Do protease	*Legionella pneumophila*	S1C	15.8
S1F	2W5E A	human astrovirus 1	3OTP A	protease Do	*Escherichia coli*	S1C	19.7
S29	2FM2 A	hepatitis C virus	3CS0 A	protease Do	*Escherichia coli*	S1C	14.4
S3	1EP5 A	Venezuelan equine encephalitis virus	3NWU B	serine protease HTRA1	*Homo sapiens*	S1C	10.9
S32	1MBM A	equine arteritis virus	3CP7 A	alkaline serine protease AL20	*Nesterenkonia aethiopica*	S1D	13.6
S39A	1ZYO A	Sesbania mosaic virus	1B0F A	protein (elastase)	*Homo sapiens*	S1A	17.8
S7	2M9P A	Dengue virus	5HMA A	MamO protease	*Magnetospirillum magneticum*	S1C	8.3
