# Supplementary material for: Structural comparison strengthens the higher-order classification of proteases related to chymotrypsin
Source: PLoS One. 2019 May 17;14(5):e0216659. doi: 10.1371/journal.pone.0216659 (PMC6524800; doi:10.1371/journal.pone.0216659)
Supplement: S1 Fig — Replicates of jackknife tests to determine the effects of dataset on overall topology of the structure-based distance tree. The protein family which member has been removed, the resulting core size and the resolution of the alignment are indicated under each tree. The colors indicate the PA clan families as in Fig 4. (PDF) [file pone.0216659.s005.pdf]

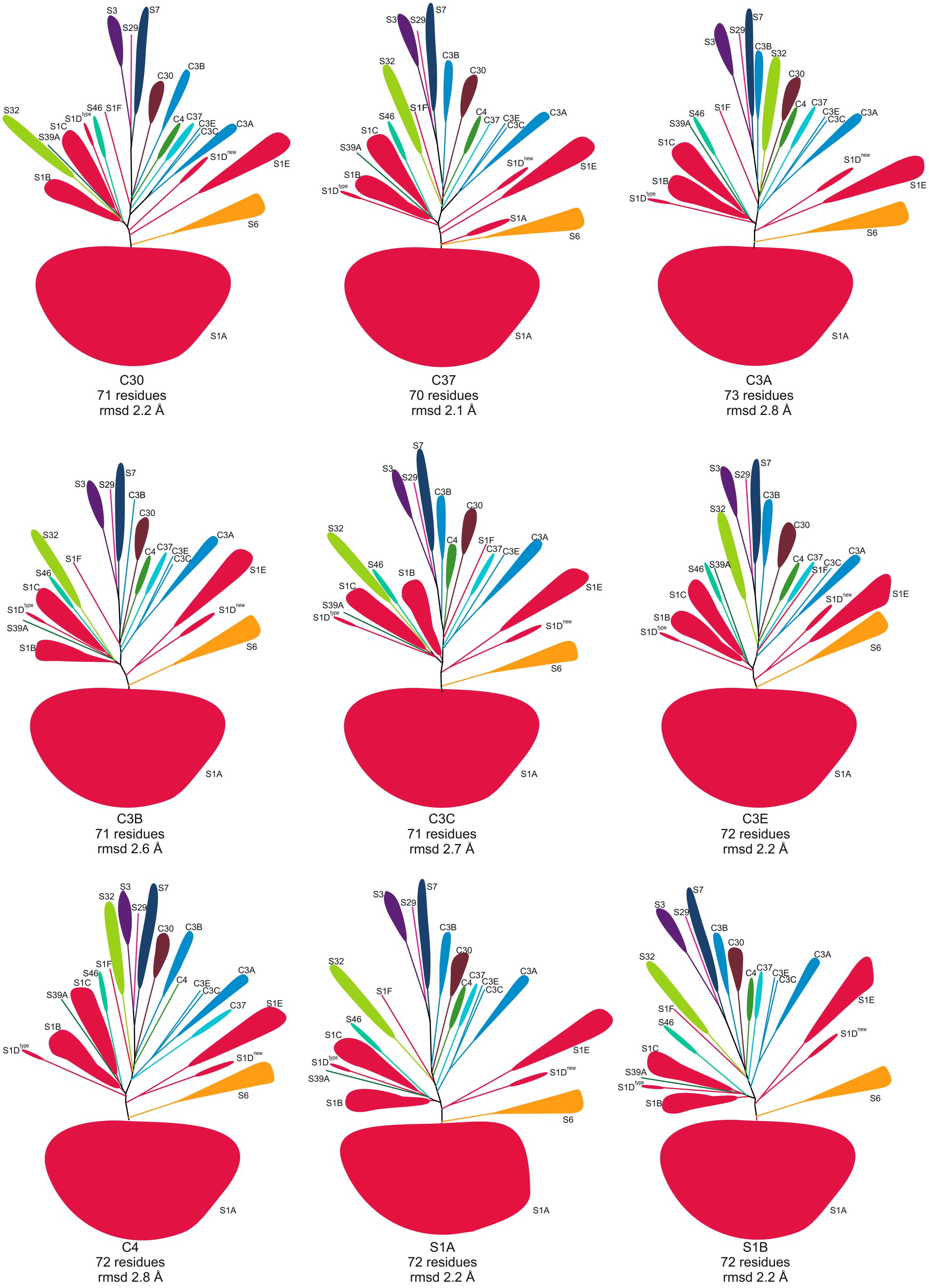
C30
71 residues
rmsd 2.2 Å
C37
70 residues
rmsd 2.1 Å
C3A
73 residues
rmsd 2.8 Å
C3B
71 residues
rmsd 2.6 Å
C3C
71 residues
rmsd 2.7 Å
C3E
72 residues
rmsd 2.2 Å
C4
72 residues
rmsd 2.8 Å
S1A
72 residues
rmsd 2.2 Å
S1B
72 residues
rmsd 2.2 Å

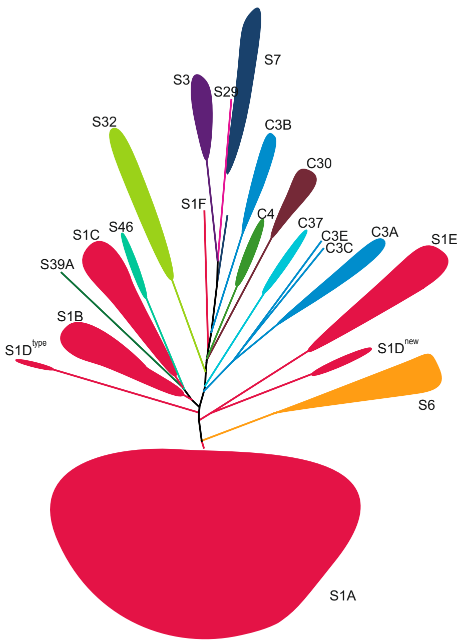

S1C
72 residues
rmsd 2.8 Å

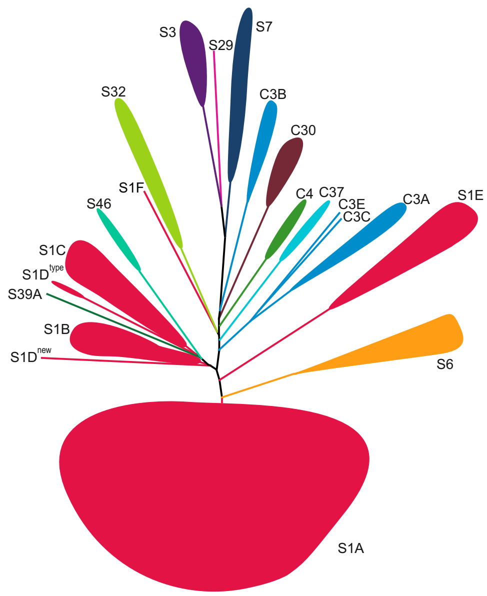

S1D
72 residues
rmsd 2.3 Å

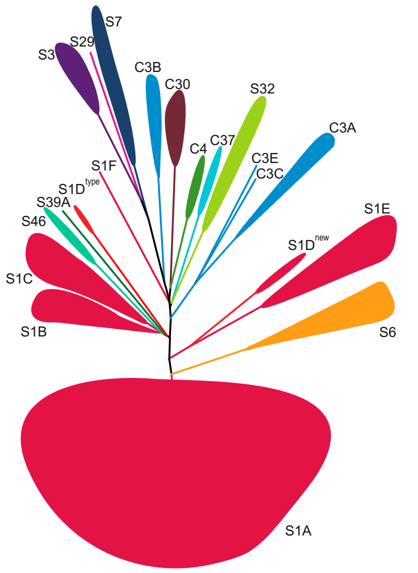

S1E
70 resiudes
rmsd 2.7 Å

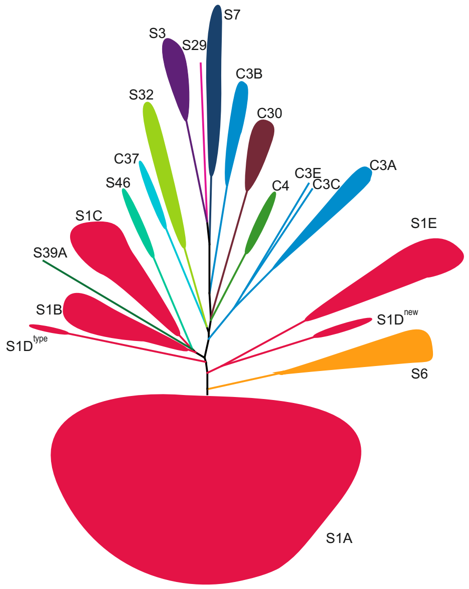

S1F
69 residues
rmsd 2.6 Å

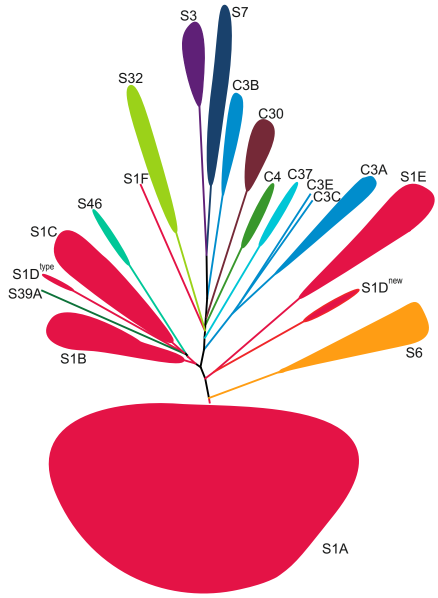

S29
74 residues
rmsd 2.7 Å

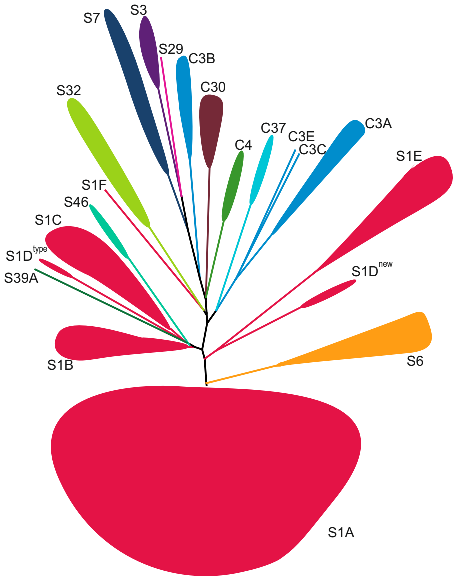

S3
72 residues
rmsd 2.4 Å

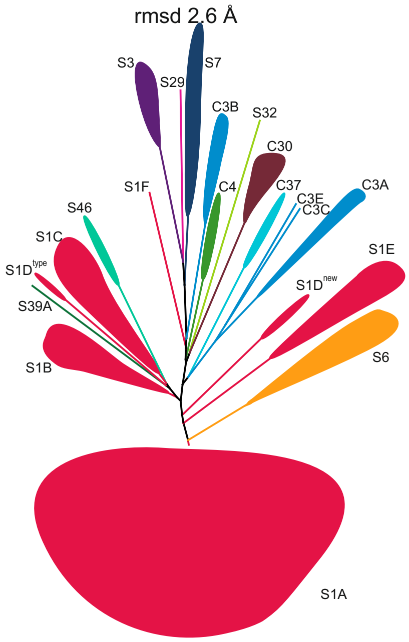

S32
73 residues
rmsd 2.9 Å

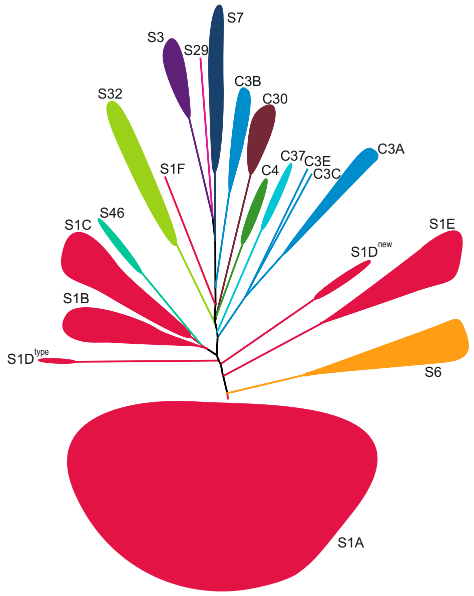

S39A
70 residues
rmsd 2.8 Å

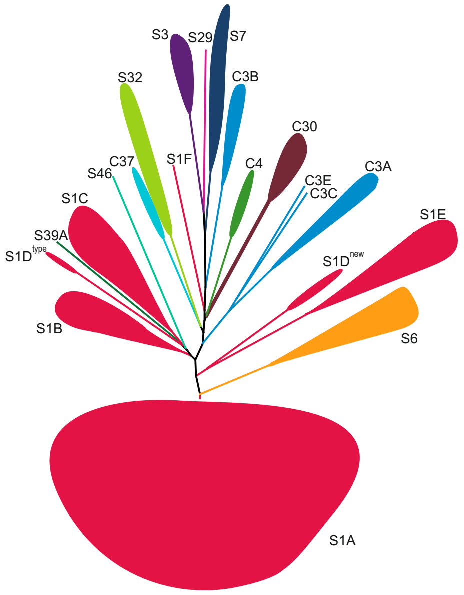

S46
71 residues
rmsd 27 Å

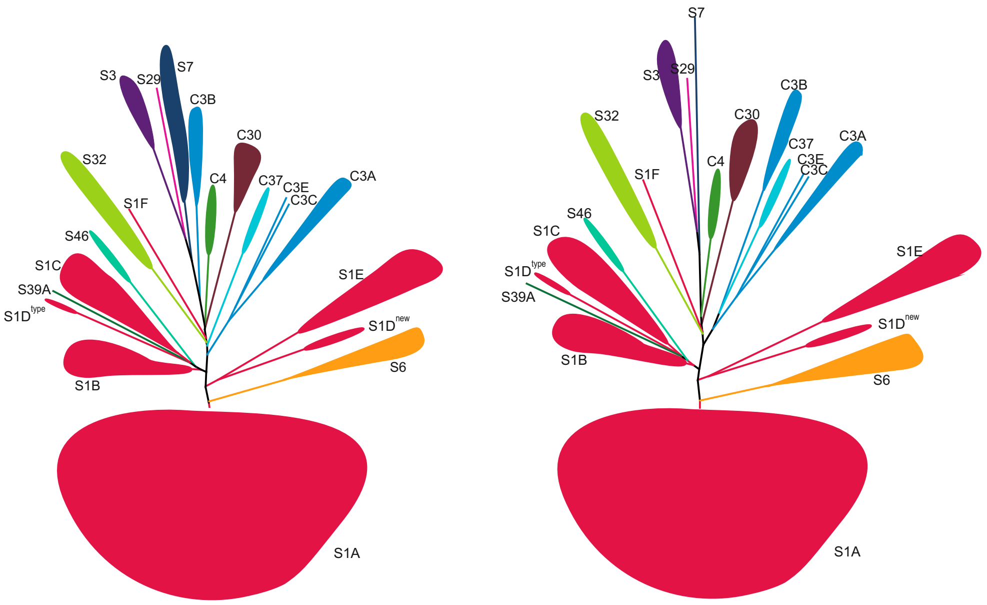

S6
72 residues
rmsd 2.7 Å

S7
75 residues
rmsd 2.4 Å

S1 Figure. Replicates of jackknife tests to determine the effects of dataset on overall topology of the structure-based distance tree. The protein family which member has been removed, the resulting core size and the resolution of the alignment are indicated under each tree. The colours indicate the PA clan families as in Figure 4.
