# Supplementary figures and images for: Structural comparison strengthens the higher-order classification of proteases related to chymotrypsin
Source: PLoS One. 2019 May 17;14(5):e0216659. doi: 10.1371/journal.pone.0216659 (PMC6524800; doi:10.1371/journal.pone.0216659)

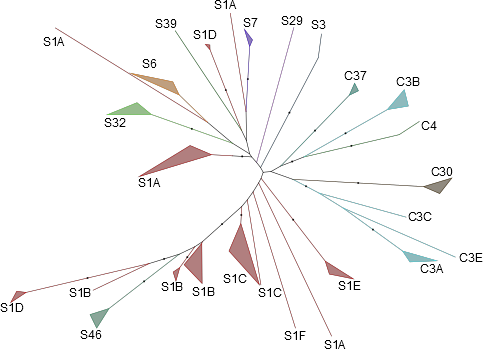

Supplement: S2 Fig — The branches are merged and colored according to the protease family as in Fig 4. The branches are marked with a black dot if the ultrafast bootstrap value is ≥0.95. (TIFF) [file pone.0216659.s006.tiff]
